# Supplementary material for: HIV-1 capsids enter the FG phase of nuclear pores like a transport receptor
Source: Nature. 2024 Jan 24;626(8000):843–51. doi: 10.1038/s41586-023-06966-w (PMC10881386; doi:10.1038/s41586-023-06966-w)
Supplement: Supplementary file 1 — Supplementary Fig. 1 and Table 1. [file 41586_2023_6966_MOESM1_ESM.docx]

SI GUIDE

**HIV-1 capsids enter the FG phase of nuclear pores like a transport receptor**

**Liran Fu^1^*, Erika N. Weiskopf^2^*, Onno Akkermans^2^*, Nicholas A. Swanson^2^, Shiya Cheng^3^, Thomas U. Schwartz^2^**, Dirk Görlich^1^****

^1^Department of Cellular Logistics, Max Planck Institute for Multidisciplinary Sciences, Göttingen, Germany; ^2^Department of Biology, Massachusetts Institute of Technology, Cambridge, MA, USA; ^3^Department of Meiosis, Max Planck Institute for Multidisciplinary Sciences, Göttingen, Germany.

*These authors contributed equally to this work;

**Co-corresponding authors:

[tus@mit.edu](mailto:tus@mit.edu)

[goerlich@mpinat.mpg.de](mailto:goerlich@mpinat.mpg.de)

**Supplementary information including:**

Supplementary Figure 1. Sequences of FG domains used for FG phase experiments.

Supplementary Table 1. Plasmids used for recombinant protein expression.

>prf.GLFG_52x12_ pSNG057 (Figs. 3-5, ED Fig.3)

G**GLFG**GNTQPATG**GLFG**GNTQPATG**GLFG**GNTQPATG**GLFG**GNTQPATG**GLFG**GNTQPATG**GLFG**GNTQPATG**GLFG**GNTQPATG**GLFG**GNTQPATG**GLFG**GNTQPATG**GLFG**GNTQPATG**GLFG**GNTQPATG**GLFG**GNTQPATG**GLFG**GNTQPATG**GLFG**GNTQPATG**GLFG**GNTQPATG**GLFG**GNTQPATG**GLFG**GNTQPATG**GLFG**GNTQPATG**GLFG**GNTQPATG**GLFG**GNTQPATG**GLFG**GNTQPATG**GLFG**GNTQPATG**GLFG**GNTQPATG**GLFG**GNTQPATG**GLFG**GNTQPATG**GLFG**GNTQPATG**GLFG**GNTQPATG**GLFG**GNTQPATG**GLFG**GNTQPATG**GLFG**GNTQPATG**GLFG**GNTQPATG**GLFG**GNTQPATG**GLFG**GNTQPATG**GLFG**GNTQPATG**GLFG**GNTQPATG**GLFG**GNTQPATG**GLFG**GNTQPATG**GLFG**GNTQPATG**GLFG**GNTQPATG**GLFG**GNTQPATG**GLFG**GNTQPATG**GLFG**GNTQPATG**GLFG**GNTQPATG**GLFG**GNTQPATG**GLFG**GNTQPATG**GLFG**GNTQPATG**GLFG**GNTQPATG**GLFG**GNTQPATG**GLFG**GNTQPATG**GLFG**GNTQPATG**GLFG**GNTQPATG**GLFG**GNTQPATS

>SLFG_52x12_ pSNG073 (Figs. 3, ED Fig. 5)

**SLFG**NTGGAPAG**SLFG**NTQTQQGG**SLFG**QPQQTQGG**SLFG**QTGATTGG**SLFG**GATNTAPG**SLFG**GGGGNPTG**SLFG**GNNNQQTG**SLFG**QGTTQTGG**SLFG**APQNNQGG**SLFG**GGTTTTGG**SLFG**ANTQTGGG**SLFG**GPSQPTTA**SLFG**SNNPTTGG**SLFG**QPANTNNG**SLFG**GQTNNQAS**SLFG**ANNQPPTN**SLFG**NNNKPQTA**SLFG**GATTTGNT**SLFG**GANNTGGG**SLFG**NNTNNPTG**SLFG**ATNPAGGG**SLFG**GGATTGGG**SLFG**GGNTQTGG**SLFG**TANTTTAG**SLFG**GGNTQPQN**SLFG**NNNTPATG**SLFG**QTNNAAPQ**SLFG**GTNNNAAS**SLFG**QKPASANGVLTKPNEKNLCYAISNGTDFCIFELALTQRKLVKAGQLKPGAQAG**SLFG**QPAQNTQG**SLFG**GGGAATTP**SLFG**GAQNNTTG**SLFG**GQNTQAGG**SLFG**APNNAAAT**SLFG**AGNANTQG**SLFG**AKPAATGG**SLFG**QPAQTQAG**SLFG**NTAQPAGG**SLFG**GATTTPGG**SLFG**GNTAATGG**SLFG**GNTQGATG**SLFG**GQQPNNQG**SLFG**NTNANTGG**SLFG**GATTTTGG**SLFG**GSTGATGG**SLFG**GASQPAAG**SLFG**GAAPQQNS**SLFG**GATAGQTG**SLFG**GATQQQGG**SLFG**QTA

>FSFG pSNG150 (Figs. 3, ED Fig. 5)

**FSFG**NTGGAPAG**FSFG**NTQTQQGGSSSGQPQQTQGG**FSFG**QTGATTGG**FSFG**GATNTAPGSSSGGGGGNPTG**FSFG**GNNNQQTG**FSFG**QGTTQTGGSSSGAPQNNQGG**FSFG**GGTTTTGG**FSFG**ANTQTGGGSSSGGPSQPTTA**FSFG**SNNPTTGG**FSFG**QPANTNNGSSSGGQTNNQAS**FSFG**ANNQPPTN**FSFG**NNNKPQTASSSGGATTTGNT**FSFG**GANNTGGG**FSFG**NNTNNPTGSSSGATNPAGGG**FSFG**GGATTGGG**FSFG**GGNTQTGGSSSGTANTTTAG**FSFG**GGNTQPQN**FSFG**NNNTPATGSSSGQTNNAAPQ**FSFG**GTNNNAAS**FSFG**QKPASANGVLTKPNEKNLCYAISNGTDFCIFELALTQRKLVKAGQLKPGAQAGSSSGQPAQNTQG**FSFG**GGGAATTP**FSFG**GAQNNTTGSSSGGQNTQAGG**FSFG**APNNAAAT**FSFG**AGNANTQGSSSGAKPAATGG**FSFG**QPAQTQAG**FSFG**NTAQPAGGSSSGGATTTPGG**FSFG**GNTAATGG**FSFG**GNTQGATGSSSGGQQPNNQG**FSFG**NTNANTGG**FSFG**GATTTTGGSSSGGSTGATGG**FSFG**GASQPAAG**FSFG**GAAPQQNSSSSGGATAGQTG**FSFG**GATQQQGG**FSFG**QTASNPGGSSSGAANATTQP**FSFG**GNNQAATS

>TbNup158 FG pHBS249 (Figs. 3, ED Fig. 5)

MSAG**FG**GG**FG**QPAATG**FG**QQPTGG**FG**QAPQGGA**FG**QVAPAATG**FG**QPSQSAVTGG**FG**QTNTGG**FG**QPAATG**FG**QPAQGAVTGG**FG**QTNTGG**FG**QPAATG**FG**QPAQSAVTGG**FG**QTNTGG**FG**QPAQGG**FG**QTAAAANA**FG**QAGPSGG**FG**QTNTGG**FG**QQSNSG**FG**QAGRGATAG**FG**QPGTG**FG**QPATGG**FG**QATSAS**PFG**QAAAGRGVGGG**FG**TAAGTVGG**FG**QPAATGG**FG**QTATTGG**FG**QPAQGAAAGG**FG**QPATGG**FG**QATSAS**PFG**QAAAGRGVGGG**FG**TAAGTVGG**FG**QPAAPGG**FG**QTATAGG**FG**QPARGAAAGG**FG**QPATGG**FG**QATSAS**PFG**QAAAGRGVGGG**FG**TAAGTVGG**FG**QPAATGG**FG**QTATTGG**FG**QPAQGANT**FG**QGTPSAGG**FG**QAGRGVTGG**FG**QTGVTGG**FG**QTATTGG**FG**QPAQGAATGG**FG**QAGRGAADG**FG**RPAQGAAAGG**FG**QPATGG**FG**QATSAS**PFG**QAAAGRGVGGG**FG**TAAGTVGG**FG**QPAAPGG**FG**QTATTGG**FG**QPGRGAAAGG**FG**QPATGG**LGLA**GGSG**FG**AAAGAGG**FG**QQSTAS

>Nup116 pHBS698 (ED Fig. 4)

M**FG**VSRGAFPSATTQ**PFG**STGST**FG**GQQQQQQPVANTSA**FG**LSQQTNTTQA**PAFG**N**FG**NQTSNS**PFG**MSGSTTANGT**PFG**QSQLTNNNASGS**IFG**GMGNNTALSAGSASVVPNSTAGTSIKPFTTFEEKDPTTGVINVFQSITCMPEYRNFSFEELRFQDYQAGRK**FG**TSQNGTGTT**FN**NPQGTTNTG**FG**IMGNNNSTTSATTG**GLFG**QKPATGM**FG**TGTGSGGG**FG**SGATNST**GLFG**SSTNLSGNSA**FG**ANKPATSG**GLFG**NTTNNPTNGTNNT**GLFG**QQNSNTNG**GLFG**QQQNS**FG**ANNVSNGGA**FG**QVNRGA**FP**QQQTQQGSGG**IFG**QSNANANGGA**FG**QQQGTGA**LFG**AKPASG**GLFG**QSAGSKA**FG**MNTNPTGTTG**GLFG**QTNQQQSGG**GLFG**QQQNSNAG**GLFG**QNNQSQNQS**GLFG**QQNSSNA**FG**QPQQQG**GLFG**SKPAG**GLFG**QQQGAST**FA**SGNAQNNS**IFG**QNNQQQQSTG**GLFG**QQNNQSQSQPG**GLFG**QTNQNNNQ**PFG**QNGLQQPQQNN**SLFG**AKPTG**FG**NT**SLFS**NSTTNQSNGISGNNLQQQSG**GLFQ**NKQQPASG**GLFG**SKPSNTVGG**GLFG**NNQVANQNNPASTSG**GLFG**SKPATG**SLFG**GTNSTAPNASSGG**IFG**SNNASNTAATTNST**GLFG**NKPVGAGASTSAG**GLFG**NNNNSSLNNSNGST**GLFG**SNNTSQSTNAG**GLFQ**NNTSTNTSGG**GLFS**QPSQSMAQSQNALQQQQQQQR

>hNup98 pHBS491 (ED Fig. 4)

MFNKS**FG**T**PFG**GGTGG**FG**TTST**FG**QNTG**FG**TTSGGA**FG**TSA**FG**SSNNTG**GLFG**NSQTKPG**GLFG**TSSFSQPATSTSTG**FGFG**TSTGTANT**LFG**TASTGTSLFSSQNNAFAQNKPTG**FG**N**FG**TSTSSG**GLFG**TTNTTSN**PFG**STSG**SLFG**PSSFTAAPTGTTIKFNPPTGTDTMVKAGVSTNISTKHQCITAMKEYESKSLEELRLEDYQANRKGPQNQVGAGTTT**GLFG**SSPATSSATGLFSSSTTNSGFAYGQNKTA**FG**TSTTG**FG**TNPG**GLFG**QQNQQTTSLFSK**PFG**QATTTQNTGFS**FG**NTSTIGQPSTNTMGS**FG**VTQASQPG**GLFG**TATNTSTGTA**FG**TGT**GLFG**QTNTG**FG**AVGST**LFG**NNKLTT**FG**SGTTSAPS**FG**TTSG**GLFGFG**TNTSGNS**IFG**SKPAPGT**LG**TG**LG**AG**FG**TA**LG**AGQA**SLFG**NNQPKIGGP**LG**TGA**FG**APGFNTTTAT**LGFG**APQAPVALTDPNASAAQQAVLQQHINSLTYS**PFG**DS

**Supplementary Figure 1. Sequences of FG domains used for FG phase experiments.**

| Protein | Plasmid | Encoding for | Figure | Reference |
| --- | --- | --- | --- | --- |
| RanQ69L_1-180_ | pTG‑A418 | His_10_-zz-Tev‑hsRanQ69L_1‑180_ | 1d | ^64^ |
| Importin β | pDG2305 | His_14_-MBP-bdSUMO-hsImportin β | 1d | ^26^ |
| IBB-EGFP | pDG2895 | His_14_-MBP-bdSUMO-hsIBB-EGFP | 1d | ^26^ |
| mCherry | pDG2442 | His_14_-bdSUMO-mCherry | 3-5,  ED 3-5 | ^26^ |
| EGFP | pSF1526 | His_14_-MBP-bdSUMO-EGFP | 1b, 3a | ^26^ |
| sinGFP4a | pDG2754 | His_14_-bdSUMO-sinGFP4a | 3, 5 | ^26^ |
| Sin_tCherry2 | pDG2804 | His_14_-bdSUMO-sintCherry2 | 4,  ED 6-7 | ^26^ |
| prf.GLFG_52x12_ | pSNG57 | His_18_- GLFG52x12 | 3-5, ED  3 | ^50^ |
| SLFG_52x12_ | pSNG73 | His_18_- SLFG52x12 | 3e, ED  5 | ^25^ |
| FSFG_52x12_ | pSNG150 | His_18_- FSFG domain | 3e ED  5 | ^25^ |
| Tb Nup158 FG | pHBS249 | His_14_ Tev FG domain | 3e, ED  5 | ^21^ |
| Sc Nup116 FG | pHBS698 | His_18_-Nup116 FG | ED  4 | ^21^ |
| hs Nup98 FG | pHBS491 | His_18_-Nup98FG | ED  4 | ^21^ |
| CA^P1A,A14C,E45C,W184A,M185A^*  (for CA hexamers) | pETDUET-OA7 | His_14_-bdSUMO-CA^P1A,A14C,E45C,W184A,M185A^ | 2, ED 1-2 | This study |
| CA^P1A,N21C,A22C^*  (for CA 40 nm capsid spheres) | pETDUET-OA43 | His_14_-bdSUMO- CA^P1A,N21C,A22C^ | 1, 3, 5, ED 3-8 | This study |
| CA^P1A,N21C,A22C^-EGFP*  (for labelling CA spheres) | pETDUET-OA45 | His_14_-bdSUMO- CA^P1A,N21C,A22C^-EGFP | 1, 3a | This study |
| CA^P1A,N21C,A22C^-sinGFP4a*  (for labelling hexamers and spheres) | pETDUET-OA87 | His_14_-bdSUMO- CA^P1A,N21C,A22C^-sinGFP4a | 3,  ED 3-6 | This study |
| CA* (for CLPs) | pETDUET-OA90 | Wild type CA | 5, ED 8 | This study |
| CA^P1A,N21C,A22C,N57A^*  (for CA 40 nm capsid spheres) | pLF741 | His_14_-bdSUMO- CA^P1A,N21C,A22C,N57A^ | 4, ED 7 | This study |
| CA^P1A,N21C,A22C,N57A^-EGFP* (for CA 40 nm capsid spheres) | pLF751 | His_14_-bdSUMO- CA^P1A,N21C,A22C,N57A^-EGFP | 4, ED 7 | This study |
| mCherry | pETDUET-AS13 | His_12_-mCherry | 5, ED 8 | This study |
| Nup50_39-130_ | pETDUET-ENW15 | Avi-bdSUMO-Nup50_39-130_-His_6_ | 2a | This study |
| Nup58_2-57_ | pETDUET-ENW03 | Avi-bdSUMO-Nup58_2-57_-His_6_ | 2a | This study |
| Nup58_523-589_ | pETDUET-ENW06 | Avi-bdSUMO - Nup58_523-589_-His_6_ | 2 | This study |
| Nup62_35-130_ | pETDUET-ENW19 | Avi-bdSUMO - Nup62_35-130_-His_6_ | 2 | This study |
| Nup98_73-156_ | pETDUET-ENW01 | Avi- bdSUMO – Nup98_73-156_-His_6_ | 2a | This study |
| Nup98_378-480_ | pETDUET-ENW12 | Avi- bdSUMO - Nup98_378-480_-His_6_ | 2 | This study |
| Nup358_1926-1985_ | pETDUET-ENW27 | Avi- bdSUMO - Nup358_1926-1985_-His_6_ | 2a | This study |
| Nup358_2509-2569_ | pETDUET-ENW28 | Avi- bdSUMO - Nup358_2509-2569_-His_6_ | 2a | This study |
| CA^N57A^ | pETDUET-ENW46 | His_14_-bdSUMO-CA^N57A^ | 2c | This study |
| CPSF6_313-327_ | pETDUET-ENW26 | Avi-bdSUMO-CPSF6_313-327-_His_6_ | ED 2 | This study |
| Nup153_1407-1423_ | pETDUET-OA21 | Avi-bdSUMO-Nup153_1407-1423_-His_6_ | ED 2 | This study |
| Importin α | pTUS1998 | His_6_-Importin α | ED 2 | This study |
| Importin β | pTUS1999 | His_6_-SUMO-Importin β | 2b, ED 2 | This study |
| Nesprin-2_1423-1745_ | pPD512 | His_14_-bdSUMO- Nesprin-2_1423-1745_ | ED 2 | This study |

**Supplementary Table 1. Plasmids used for recombinant protein expression.**
