# Supplementary material for: HIV-1 capsids enter the FG phase of nuclear pores like a transport receptor
Source: Nature. 2024 Jan 24;626(8000):843–51. doi: 10.1038/s41586-023-06966-w (PMC10881386; doi:10.1038/s41586-023-06966-w)
Supplement: Supplementary file 2 — Reporting Summary [file 41586_2023_6966_MOESM2_ESM.pdf]

nature portfolio

Corresponding author(s): Dirk Görlich, Thomas Schwartz

Last updated by author(s): 2023 10 27

Reporting Summary

Nature Portfolio wishes to improve the reproducibility of the work that we publish. This form provides structure for consistency and transparency in reporting. For further information on Nature Portfolio policies, see our Editorial Policies and the Editorial Policy Checklist.

Statistics

For all statistical analyses, confirm that the following items are present in the figure legend, table legend, main text, or Methods section.

n/a	Confirmed	
☐	☒	The exact sample size (*n*) for each experimental group/condition, given as a discrete number and unit of measurement
☒	☐	A statement on whether measurements were taken from distinct samples or whether the same sample was measured repeatedly
☒	☐	The statistical test(s) used AND whether they are one- or two-sided<br>*Only common tests should be described solely by name; describe more complex techniques in the Methods section.*
☒	☐	A description of all covariates tested
☒	☐	A description of any assumptions or corrections, such as tests of normality and adjustment for multiple comparisons
☐	☒	A full description of the statistical parameters including central tendency (e.g. means) or other basic estimates (e.g. regression coefficient) AND variation (e.g. standard deviation) or associated estimates of uncertainty (e.g. confidence intervals)
☒	☐	For null hypothesis testing, the test statistic (e.g. *F*, *t*, *r*) with confidence intervals, effect sizes, degrees of freedom and *P* value noted<br>*Give P values as exact values whenever suitable.*
☒	☐	For Bayesian analysis, information on the choice of priors and Markov chain Monte Carlo settings
☒	☐	For hierarchical and complex designs, identification of the appropriate level for tests and full reporting of outcomes
☒	☐	Estimates of effect sizes (e.g. Cohen's *d*, Pearson's *r*), indicating how they were calculated

Our web collection on statistics for biologists contains articles on many of the points above.

Software and code

Policy information about availability of computer code

Data collection	was using commercial instruments and the included software (FEI Tecnai G2 Spirit TWIN 120 kV transmission electron microscope, Leica SP8 confocal laser scanning microscope, Zeiss LSM 880 confocal laser scanning microscopes, forteBIO OctetRED96)
Data analysis	Instrumental software, FIJI 2.9.0 (Schindelin et al., 2012 Fiji: an open-source platform for biological-image analysis. Nat Methods 9, 676-682) and GraphPad Prism 9.5.1 (GraphPad Software, Boston, Massachusetts USA, www.graphpad.com)

For manuscripts utilizing custom algorithms or software that are central to the research but not yet described in published literature, software must be made available to editors and reviewers. We strongly encourage code deposition in a community repository (e.g. GitHub). See the Nature Portfolio guidelines for submitting code & software for further information.

Data

Policy information about availability of data

All manuscripts must include a data availability statement. This statement should provide the following information, where applicable:

- Accession codes, unique identifiers, or web links for publicly available datasets
- A description of any restrictions on data availability
- For clinical datasets or third party data, please ensure that the statement adheres to our policy

All data that are described in the manuscript are also explicitly shown in the figures

Research involving human participants, their data, or biological material

Policy information about studies with human participants or human data. See also policy information about sex, gender (identity/presentation), and sexual orientation and race, ethnicity and racism.

Reporting on sex and gender	not applicable
Reporting on race, ethnicity, or other socially relevant groupings	not applicable
Population characteristics	not applicable
Recruitment	not applicable
Ethics oversight	not applicable

Note that full information on the approval of the study protocol must also be provided in the manuscript.

Field-specific reporting

Please select the one below that is the best fit for your research. If you are not sure, read the appropriate sections before making your selection.

☒ Life sciences ☐ Behavioural & social sciences ☐ Ecological, evolutionary & environmental sciences

For a reference copy of the document with all sections, see nature.com/documents/nr-reporting-summary-flat.pdf

Life sciences study design

All studies must disclose on these points even when the disclosure is negative.

Sample size	No statistical methods were used to predetermine sample size. The effects described here are obvious, reproducible, clear, and homogeneous enough that no statistical methods were required to extract the trait. For example, all inspected cells on a coverslip showed NPC-targeting of capsids, and all performed FG-phase experiments showed extremely strong intra-phase capsid accumulation (to a partition coefficient of ≥100 which is >1000 times higher than GFP or mCherry alone). For partition coefficient calcualtion, FG particles numbers >10 is enough to cover the effects.
Data exclusions	None.
Replication	Seven times for Figure 1b, three times for Figure 1c and 1d; four times for Figure 3a and 3e, thirteen times for Figure 3b; three times for Figure 4; five times for Figure 5. Four times for Extended Data Figure 3 and 4, three times for Extended Data Figure 5. All attempts for replication were successful.
Randomization	The allocations of FG particles/HeLa-Kyoto cells into wells of plates for test were random. Mouse oocytes were randomly split into different groups.
Blinding	Investigators were not blinded to allocation during the experiments and analysis, as each experiment was conducted by a single investigator.

Reporting for specific materials, systems and methods

We require information from authors about some types of materials, experimental systems and methods used in many studies. Here, indicate whether each material, system or method listed is relevant to your study. If you are not sure if a list item applies to your research, read the appropriate section before selecting a response.

Materials & experimental systems

n/a	Involved in the study	
☐	☒	Antibodies
☐	☒	Eukaryotic cell lines
☒	☐	Palaeontology and archaeology
☒	☐	Animals and other organisms
☒	☐	Clinical data
☒	☐	Dual use research of concern
☒	☐	Plants

Methods

n/a	Involved in the study	
☒	☐	ChIP-seq
☒	☐	Flow cytometry
☒	☐	MRI-based neuroimaging

Antibodies

Antibodies used	anti-Nup133 nanobody as described in: Colom MS, Fu Z, Güttler T, Trakhanov S, Srinivasan V, Gregor K, Pleiner T, Görlich D (2023)
Validation	We (the Görlich lab) are the original source and the publication cited above includes the description and in-depth-validation.

Eukaryotic cell lines

Policy information about cell lines and Sex and Gender in Research

Cell line source(s)	HeLa-Kyoto
Authentication	Commercially availabe (ECACC;RRID:CVCL_1922)
Mycoplasma contamination	negative
Commonly misidentified lines (See ICLAC register)	no commonly misidentified cell line was used in the study,
